# Cystic Fibrosis in the Era of Precision Medicine: Transformative Progress and Persistent Challenges

**DOI:** 10.3390/jcm15062443

**Published:** 2026-03-23

**Authors:** Michal Gur, Lea Bentur

**Affiliations:** 1 Pediatric Pulmonary Institute and CF Center, Ruth Rappaport Children’s Hospital, Rambam Medical Center, P.O. Box 9602, Haifa 3109601, Israel; 2 Faculty of Medicine, The Technion—Israel Institute of Technology, Haifa 3525422, Israel

Over the past decade, the therapeutic landscape of cystic fibrosis (CF) has undergone a historic transformation; once considered a progressive, life-limiting multisystem disease treated exclusively with symptomatic therapies, CF has entered the era of mutation-specific treatment, for Contribution 1. The clinical implementation of highly effective CFTR modulators (CFTRm), especially elaxacaftor/tezacaftor/ivacaftor (ETI), represent one of the most remarkable achievements in precision medicine.

CFTRm have fundamentally altered disease trajectory for eligible patients. Improvements in lung function, nutritional status, pulmonary exacerbation rates, quality of life, with reductions in hospitalizations and transplant referrals, have reshaped clinical expectations. Early registry data suggest survival projections approaching those of the general population. Yet, this therapeutic revolution has not eliminated the complexity of CF, for Contribution 1, and significant questions remained regarding modulator therapy.

**Earlier administration**: Could intrauterine, or immediate postnatal CFTR correction, prevent irreversible pathology? Recently, isolated case reports highlighted the potential to prevent meconium ileus with ETI given prenatally, for Contribution 2. Early structural abnormalities have been documented in infancy, raising the possibility that intervention may need to begin even earlier than currently approved.

**Non-responsive mutations and the unmet need of nonsense variants**

Approximately 10% of individuals with CF do not benefit from current CFTRm. Among them are patients carrying nonsense mutations such as W1282X, in whom truncated protein production limits modulator efficacy. Strategies under investigation include read-through compounds, mRNA therapy, gene editing, gene replacement, and RNA-based correction.

**Formulation, delivery, and next-generation modulators**

Optimization of drug delivery, alternative formulations, improved pharmacokinetics, and next-generation CFTR correctors and potentiators are actively being developed.

**Cost, accessibility, and social inequity**

Perhaps the most urgent global challenge is inequitable access. CFTRm are among the most expensive chronic therapies in modern medicine. In many regions, access remains restricted or unavailable. Even within high-income countries, disparities persist based on socioeconomic status, insurance systems, and healthcare infrastructure.

***Beyond CFTR: The Multi-organ Reality of Cystic Fibrosis***

Importantly, CF remains a complex multisystem disorder. While pulmonary disease has been the primary therapeutic target, CF-related diabetes, liver disease, gastrointestinal dysfunction, bone health, mental health, fertility, and psychosocial burden continue to demand comprehensive care.

Moreover, individuals who begin modulator therapy after established structural lung disease may still experience bronchiectasis progression, chronic infection, and inflammatory sequelae. Airway microbiology is evolving in the modulator era, and long-term implications remain unclear.

Thus, CF care continues to require a holistic approach. Multidisciplinary teams, including pulmonologists, gastroenterologists, endocrinologists, dietitians, physiotherapists, psychologists, social workers, genetic counselors, and specialized nurses, remain central to optimal outcomes. Precision pharmacotherapy does not replace integrated care; rather, it elevates the need for coordinated long-term management strategies.

***In This Special Issue***

This Special Issue brings together six contributions that reflect CF research—from molecular innovation to clinical implementation and health systems considerations.

As mentioned earlier, restoration of CFTR function does not equate to disease resolution. Treatment demands precision monitoring, innovative anti-infective strategies, technological solutions for chronic care, and sustained vigilance regarding long-term safety.

Airway clearance therapy (ACT) has remained one of the fundamental treatments in CF. Even in patients on CFTRm, particularly those with established bronchiectasis or chronic infection, ACT may improve the vicious cycle of airway obstruction, inflammation and infection. Helper et al. examined a novel device—the LibAirty™ airway clearance system; mucus clearance achieved was comparable to expert physiotherapists and superior to oscillatory devices, addressing workforce shortages in respiratory care, for Contribution 3.

In an era where traditional clinical trajectories are being reshaped by modulators, accessible biomarkers for risk assessment are still needed. The longitudinal work by Goldberg et al. repositions serum IgG as a valuable biomarker, identifying a hyper-inflammatory phenotype associated with rapid progression to advanced lung disease. People with CF (pwCF) with baseline IgG Z-scores above the 97.5th percentile were significantly more likely to reach clinical end stage (including FEV1 < 40%, referral for lung transplantation, or death), for Contribution 4. Their findings remind us that biological heterogeneity persists despite genetic correction.

While improving CF lung disease with CFTRm, airway infection remains a significant challenge, with emerging antibiotic resistance posing an even more demanding obstacle. The translational study by Heching et al. introduces OMN51, a membrane-targeting antimicrobial peptide with potent activity against multi-drug-resistant Pseudomonas aeruginosa, for Contribution 5. By bypassing classical resistance pathways, this work represents a potential paradigm shift in CF infectious disease management, offering hope against pathogens that increasingly evade conventional antibiotics.

Similarly, the 14-year analysis by Bar-On et al. clarifies the pathogenic significance of Achromobacter xylosoxidans. By demonstrating its association with accelerated lung function decline and increased exacerbation burden, the study challenges any residual perception of this organism as a benign colonizer, for Contribution 6. Microbiological complexity continues to evolve, particularly in patients with advanced structural lung disease.

The formidable challenge of non-tuberculous mycobacterial (NTM) infection is addressed by Basher et al., whose comprehensive exploration of Mycobacterium abscessus (Mab) pathogenesis highlights the intricate host–pathogen interplay within the CF airway. Mab pulmonary disease leads to progressive lung dysfunction and remains a significant therapeutic challenge due to intrinsic and acquired antibiotic resistance, for Contribution 7. Their synthesis underscores why eradication remains elusive and why innovative approaches—ranging from host-directed therapies to bacteriophage treatment—must be pursued with urgency.

Finally, Lucca et al. provide critical five-year real-world safety data on ETI therapy. Their findings confirm overall tolerability but highlight important psychiatric, hepatic, and musculoskeletal adverse events that require long-term monitoring and individualized dose adjustment, for Contribution 8. As treatment begins earlier in life and extends across decades, longitudinal pharmacovigilance becomes essential.

Taken together, the manuscripts in this Special Issue illustrate that CF remains a multi-organ, multi-dimensional disease. Mutation-specific therapies have revolutionized care, yet persistent inflammation, chronic infection, structural lung damage, emerging pathogens, antimicrobial resistance, and treatment-related adverse events continue to shape outcomes.

The future of CF care will therefore depend not only on correcting CFTR dysfunction but on integrating precision biomarkers, innovative anti-infective agents, advanced airway clearance technologies, host-directed strategies, and rigorous long-term safety surveillance within a multidisciplinary framework.

